# The actinobacterial WhiB‐like (Wbl) family of transcription factors

**DOI:** 10.1111/mmi.14117

**Published:** 2018-10-25

**Authors:** Matthew J. Bush

**Affiliations:** ^1^ Department of Molecular Microbiology John Innes Centre Norwich Research Park, Norwich NR4 7UH UK

## Abstract

The WhiB‐like (Wbl) family of proteins are exclusively found in Actinobacteria. Wbls have been shown to play key roles in virulence and antibiotic resistance in *Mycobacteria *and *Corynebacteria*, reflecting their importance during infection by the human pathogens *Mycobacterium tuberculosis, Mycobacterium leprae* and *Corynebacterium diphtheriae*. In the antibiotic‐producing *Streptomyces*, several Wbls have important roles in the regulation of morphological differentiation, including WhiB, a protein that controls the initiation of sporulation septation and the founding member of the Wbl family. In recent years, genome sequencing has revealed the prevalence of Wbl paralogues in species throughout the Actinobacteria. Wbl proteins are small (generally ~80–140 residues) and each contains four invariant cysteine residues that bind an O_2_‐ and NO‐sensitive [4Fe–4S] cluster, raising the question as to how they can maintain distinct cellular functions within a given species. Despite their discovery over 25 years ago, the Wbl protein family has largely remained enigmatic. Here I summarise recent research in *Mycobacteria, Corynebacteria *and *Streptomyces *that sheds light on the biochemical function of Wbls as transcription factors and as potential sensors of O_2_ and NO. I suggest that Wbl evolution has created diversity in protein–protein interactions, [4Fe–4S] cluster‐sensitivity and the ability to bind DNA.

## Introduction

The WhiB‐like (Wbl) family of proteins, of which WhiB is the founding member, are exclusively found in Actinobacteria. Following the initial characterisation of WhiB in *Streptomyces coelicolor* (*Sc*) (Chater, 1972; Davis and Chater, 1992), multiple paralogues were identified first in *Mycobacteria* (Cole *et al.*, 1998) and then in other species of *Streptomyces *(Soliveri *et al.*, 2000). Since then, genome sequencing has revealed the prevalence of Wbl paralogues throughout the phylum (Table 1). In *S. coelicolor*, there are 14 Wbl proteins with 11 encoded on the chromosome and three encoded on the large linear plasmid, SCP1 (Bentley *et al.*, 2002; 2004). In *Mycobacteriun* *tuberculosis* (*Mtb*) there are seven paralogues and in *Corynebacterium glutamicum* (*Cg*) there are four.

**Table 1:**
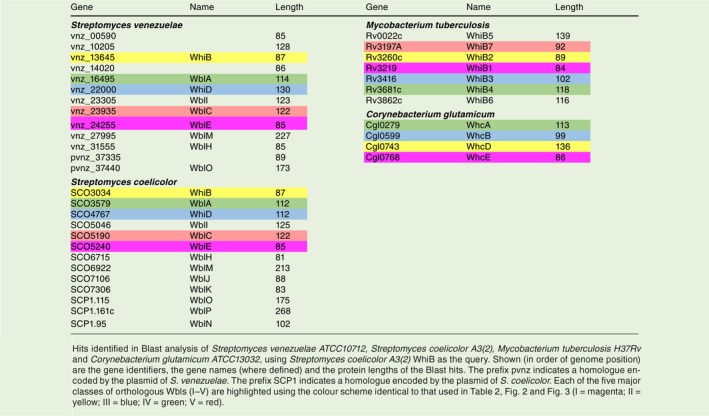
WhiB homologs in Actinobacteria.

**Figure 1:**
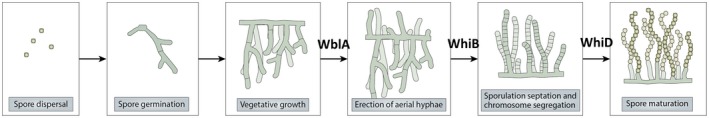
Regulation of *Streptomyces* differentiation by WhiB‐like proteins. 3 Wbl proteins have been shown to regulate separate stages in the developmental life‐cycle. WblA is involved in the early stages of aerial hyphae formation, WhiB controls the initiation of sporulation septation and WhiD is involved in the later stages of spore formation.

*Streptomyces *are prolific producers of antibiotics and their production is both temporally and genetically linked to a complex process of differentiation (Fig. 1). Like other members of the Actinobacteria, *Streptomyces *employ a polar mode of growth (Flärdh *et al.*, 2012), which, following germination and coupled to branching, establishes a vegetative mycelium. Upon nutrient depletion, *Streptomyces *initiate a series of complex developmental transitions to generate stress‐resistant spores (Flärdh and Buttner, 2009; Bush *et al.*, 2015). Non‐branching aerial hyphae grow upwards away from the colony surface before multiple division septa divide the multi‐genomic compartments into uni‐genomic spores. Several *wbl* mutants show defects in either aerial hyphae formation or sporulation, reflecting the importance of Wbls in development (Fig. 1). Recently, a distinct ‘exploratory’ growth mode has been identified in which non‐branching vegetative hyphae of *Streptomyces *rapidly extend across surfaces, in response to glucose starvation and the presence of a volatile compound, trimethylamine, that raises pH (Jones *et al.*, 2017; Jones and Elliot, 2018). However, *S. venezuelae *mutants lacking the Wbls WhiB or WhiD are still competent to explore, showing that they do not regulate this process (Jones *et al.*, 2017).

Like *Streptomyces, Mycobacteria *and *Corynebacteria* grow via the insertion of peptidoglycan at the poles. Cell division occurs as in other rod‐shaped bacteria, with the cell division machinery assembling at mid‐cell before a final mechanically driven ‘snapping’ event that separates the daughter cells (Zhou *et al.*, 2016). The cell wall in *Mycobacteria *and *Corynebacteria *is particularly complex with an outer membrane consisting of mycolic acids that are covalently linked to the peptidoglycan (Hett and Rubin, 2008; Donovan and Bramkamp, 2014). This complexity contributes significantly to the resistance of both genera to antibiotics and other stresses, underlying the difficulty in the treatment of infections by the human pathogens *M. tuberculosis*, *Mycobacterium leprae *and *C. diphtheriae*. Significantly, Wbls have been shown to play key roles in virulence and antibiotic resistance, especially in *Mycobacteria*, further highlighting the value of research into this protein family (Morris *et al.*, 2005; Singh *et al.*, 2009; Burian *et al.*, 2012; Chawla *et al.*, 2012; Mehta *et al.*, 2016).

**Figure 2:**
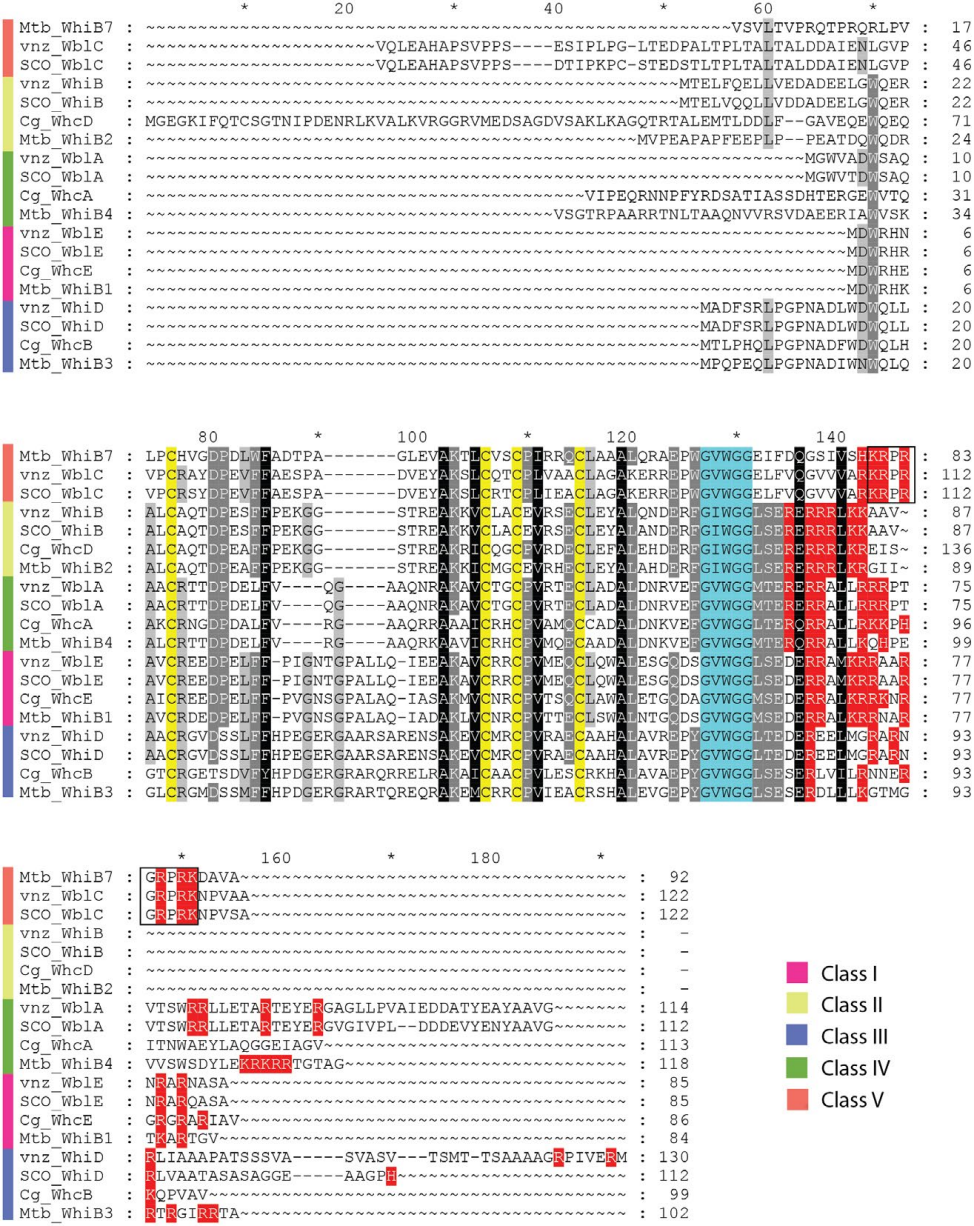
Protein sequence alignment of Wbl‐family members. Shown is an amino acid sequence alignments of Wbl proteins from *Streptomyces venezuelae* *ATCC10712 *(vnz)*, Streptomyces coelicolor A3(2) *(SCO)*, Mycobacterium tuberculosis H37Rv *(Mtb) and* Corynebacterium glutamicum* *ATCC13032 *(Cg). The alignment is restricted to members of the five classes of orthologous proteins (I–V) that have been the subject of significant study in Actinobacteria. As shown by the key, these classes are highlighted with the colour scheme identical to that used in Table 1, Table 2 and Fig. 3. The four invariant cysteines (C) are highlighted in yellow and the G(V/I)WGG motif is highlighted in turquoise. Positively charged residues that may facilitate DNA binding or interaction with DNA are highlighted in red. The ‘KRPRGRPRK’ AT‐hook motif, present in the WblC/WhiB7 family, is boxed.

**Figure 3:**
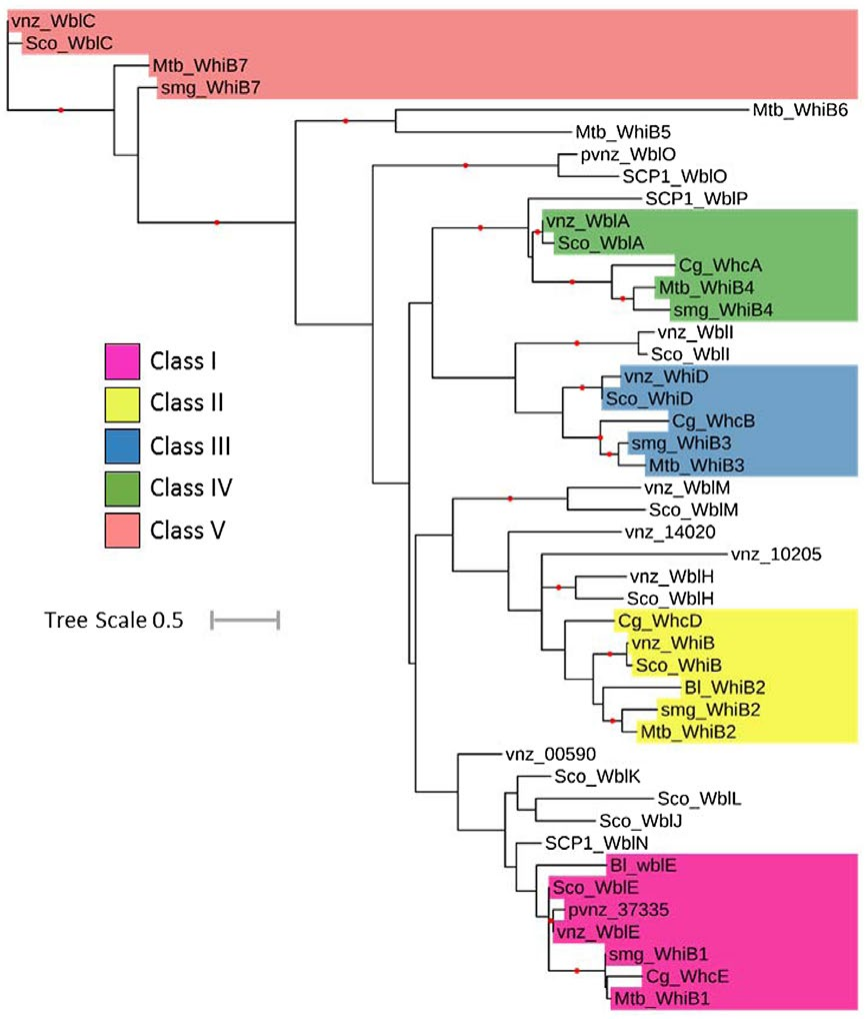
Phylogenetic tree of selected WhiB‐like proteins. Homologues were identified by Blast analysis using *S. coelicolor *A3(2) WhiB as the query. Searches were made against *Streptomyces venezuelae *ATCC10712 (vnz)*, Streptomyces coelicolor *A3(2) (Sco)*, Mycobacterium tuberculosis *H37Rv (Mtb), *Mycobacterium smegmatis* *str. MC2 155* (smg), *Bifidobacterium longum *NCC2705 (Bl) and* Corynebacterium glutamicum *ATCC13032 (Cg). Amino acid sequences were aligned using CLUSTAL (Higgins & Sharp, 1988), and the resulting multiple sequence alignment was manually edited to ensure the alignment of four cysteine residues that are known to be completely conserved among Wbls. The resulting multiple sequence alignment was used to build a maximum likelihood phylogeny with 100 bootstrap replicates by PhyML 3.0 (Guindon *et al.*, 2010) using the online server available at https://www.atgc-montpellier.fr/phyml/. WAG+G+I+F was selected as the best‐fit model based on a Smart Model Selection (Lefort *et al.*, 2017) analysis of the multiple sequence alignment. iTOL software (https://itol.embl.de/) was used to generate the tree as displayed. Bootstrap values > 50% are indicated at their respective nodes by red dots (based on 100 replicates). The prefix pvnz indicates a homologue encoded by the plasmid of *S. venezuelae*. The prefix SCP1 indicates a homologue encoded by the plasmid of *S. coelicolor*. Tree Scale shown is 0.5 substitutions per site. As shown by the key, highlighted are members of the five major classes of orthologous Wbls (I–V) with the colour scheme identical to that used in Table 1, Table 2 and Fig. 2.

Despite their discovery over 25 years ago, the Wbl protein family has largely remained enigmatic. Wbl proteins are relatively small, typically ranging from 81–139 residues in length (apart from the larger WblM, WblO and WblP proteins; Table 1). A unifying feature of all Wbls is the occurrence of four invariant cysteine residues that bind a [4Fe–4S] cluster (Fig. 2). The only other universally conserved sequence is a five residue (G[V/I]WGG) motif (Fig. 2), located at one end of a predicted loop that follows the last conserved cysteine. Based on predictions of conserved structural regions, this loop has previously been suggested to be a ‘prime candidate’ for interaction with another cellular component (Soliveri *et al.*, 2000). More recently, research using model species in *Streptomyces, Mycobacteria *and *Corynebacteria *has revealed the central role of Wbls in the biology of Actinobacteria and shed light on their biochemical function as transcription factors and as potential sensors of O_2_ or nitic oxide (NO). Here I outline the findings of research into the five major classes of orthologous Wbls (Table 2 and Fig. 3) and summarise their contribution to our understanding of this important protein family.

**Table 2:**
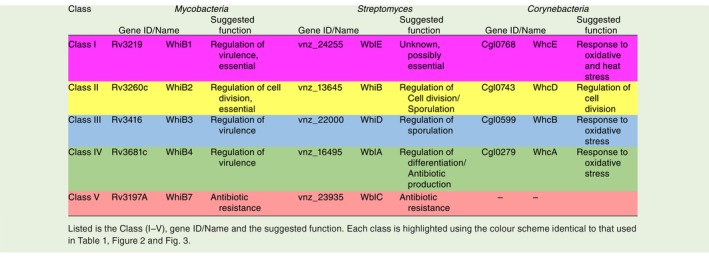
The five classes of orthologous proteins in *Mycobacteria, Streptomyces *and *Corynebacteria*.

## Class I (WhiB1/WblE/WhcE)

In *M. tuberculosis*, the *whiB1 *gene is essential (Smith *et al.*, 2010). In *Streptomyces*, there have been two contrasting studies, one suggesting that the *wblE *gene may similarly be essential (Fowler‐Goldsworthy *et al.*, 2011), and a second reporting that a Δ*wblE *mutant can be constructed and has no obvious phenotype (Homerová *et al.*, 2003). Further study is therefore required to determine the importance of WblE. In *C. glutamicum*, deletion of the orthologous gene, *whcE* leads to an increased sensitivity to heat and oxidative stress (Kim *et al.*, 2005). Oxidised WhcE*_Cg_* interacts with the efflux pump SpiE (Stress Protein Interacting with WhcE) and expression of the genes encoding both proteins increases under heat and oxidative stress, suggesting that WhcE*_Cg_* and SpiE collectively contribute to mediating the cellular response under these conditions (Park *et al.*, 2016).

**Figure 4:**
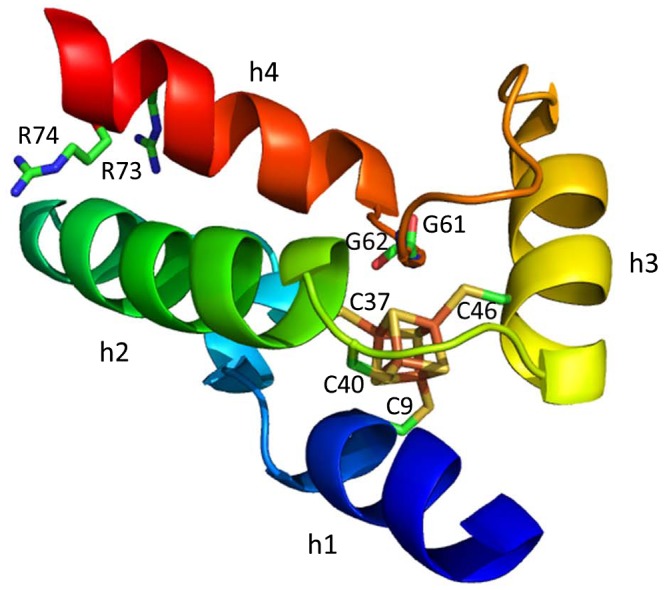
The Structure of WhiB1 from *Mycobacterium tuberculosis* (Kudhair *et al.*, 2017). Labelled are the four conserved Cysteine residues (C9, C37, C40 and C46), two Glycines (G61 and G62) that are part of the conserved G(V/I)WGG turn and the C‐terminal Arginine residues (R73 and R74) that are proposed to interact with DNA in *M. tuberculosis*. Helices are labelled (h1‐h4). PDB ID:5oay.

The [4Fe–4S] cluster of *M. tuberculosis* WhiB1 appears particularly O_2_‐stable (Smith *et al.*, 2010; Crack *et al.*, 2011), facilitating the application of NMR to generate the first structure of a WhiB‐like protein (Kudhair *et al.*, 2017). This model indicates the [4Fe–4S] cluster holds three α‐helices in place, generating a compact structure (Fig. 4). The four invariant cysteines coordinate the cluster, with the loop between helix 3 and 4 (that includes the terminal two glycine residues of the conserved ‘middle domain’ G[V/I]WGG) running along one face of the cluster. A fourth, C‐terminal α‐helix includes positively charged residues, conserved to varying levels in other Wbl homologues (Fig. 2), which have been suggested to facilitate DNA‐binding. These include two arginine residues (R73 and R74) that are required for DNA‐binding by apo‐WhiB1*_Mtb_* *in vitro *(Smith *et al.*, 2012).

Experiments with protein expressed and purified from *E. coli* before anaerobic reconstitution to re‐assemble the cluster *in vitro *(Smith *et al.*, 2010) and experiments with native cluster‐containing protein expressed and purified from *M. smegmatis* (Kudhair *et al.*, 2017) revealed that the [4Fe–4S] cluster of WhiB1*_Mtb_* reacts with eight molecules of NO. Identical observations have been made for the Wbl paralogue WhiD from *S. coelicolor* (see below; Crack *et al.*, 2011), suggesting that this may be a conserved property of WhiB‐like proteins. The apparent NO‐sensitivity of Wbls, including WhiB1*_Mtb_*, may be particularly significant in *Mycobacteria *where NO produced by lung macrophages is a major determinant of infection by *M. tuberculosis*. The significance of the NO‐sensing capability of Wbls (including WblE) in *Streptomyces *has yet to be investigated. *In vitro, *holo‐WhiB1*_Mtb_* cannot bind to DNA but the apo‐form is able to bind and repress transcription from its own promoter (Smith *et al.*, 2010) as well as the promoters of *groEL2 *(encoding an essential chaperonin; Stapleton *et al.*, 2012) and the *espA *operon that encodes for protein components required for ESX‐1 secretion, a major virulence factor of *M. tuberculosis *(Kudhair *et al.*, 2017). Since holo‐WhiB1 is unable to bind to DNA *in vitro*, the structural model would suggest that cluster disassembly (e.g. via nitrosylation) is required to make the C‐terminal helix available for interaction with DNA. In support of this, NMR of apo‐WhiB1*_Mtb_*, prepared via nitrosylation, suggests a substantial conformational change upon addition of NO (Kudhair *et al.*, 2017).

As has been shown for other Wbl paralogues in *M. tuberculosis *(Steyn *et al.*, 2002; Burian *et al.*, 2013), WhiB1*_Mtb_* interacts with the primary sigma factor, SigA (equivalent to σ^70^ in *E. coli*), via region 4 of the sigma (Kudhair *et al.*, 2017). The conserved G[V/I]WGG motif has been speculated to be part of loop in Wbls that facilitates protein‐protein interaction (Soliveri *et al.*, 2000), and therefore, the conformation of the loop between helix 3 and helix 4 that runs across the face of [4Fe–4S] cluster (Fig. 4) may be important for this interaction. In line with this, the WhiB1*_Mtb_*‐SigA interaction is dependent upon the presence of the [4Fe–4S] cluster and is disrupted under conditions of iron limitation or nitrosative stress (Kudhair *et al.*, 2017). Since only apo‐WhiB1 has been shown to bind DNA, the significance of the interaction between holo‐WhiB1 and SigA requires further study. One possibility is that holo‐WhiB1*_Mtb_* can activate SigA‐dependent transcription in a manner that is distinct from apo‐WhiB1*_Mtb_*‐mediated gene repression (Kudhair *et al.*, 2017).

## Class II (WhiB2/WhiB/WhcD)

*Streptomyces* WhiB was the first identified Wbl family member (Chater, 1972). WhiB has since been shown to co‐control the cessation of aerial growth and the initiation of sporulation with an unrelated transcription factor, WhiA. *whiA *and *whiB *mutants of *Streptomyces *fail to halt aerial growth and do not initiate sporulation septation, giving long, extended aerial hyphae and no spores (Aínsa *et al.*, 2000; Soliveri *et al.*, 2000; Bush *et al.*, 2013; Bush *et al.*, 2016). These identical phenotypes led to the suggestion that WhiA and WhiB might function together to control a distinct pathway in the regulatory network underlying differentiation. Subsequently, *in vivo *ChIP‐seq in *S. venezuelae *(*Sv*) coupled with transcriptional profiling of *whiA* and *whiB* mutants revealed that WhiA and WhiB have identical regulons, binding to the promoter regions of the same ~250 transcriptional units, including those involved in cell division, e.g. *ftsZ, ftsW *(Bush *et al.*, 2013; Bush *et al.*, 2016)*.* These WhiB ChIP‐seq results strongly suggests that WhiB, and therefore, probably all Wbls, function to regulate transcription *in vivo*. WhiA promoter binding *in vivo *was not observed in a *whiB *mutant, or *vice versa*, suggesting that the binding of WhiA and WhiB to DNA is co‐dependent. Furthermore, substitution of the invariant cysteine residues of WhiB prevents DNA binding *in vivo* by WhiB and, in‐turn, WhiA, demonstrating the importance of the [4Fe–4S] cluster in WhiB function (Bush *et al.*, 2016).

The structure of WhiA consists of two distinct domains. The large N‐terminal domain is distantly related to the LAGLIDADG eukaryotic family of homing endonucleases but is non‐catalytic (it lacks key conserved residues) and cannot bind to DNA, meaning its function in WhiA is unknown (Kaiser *et al.*, 2009; Kaiser and Stoddard, 2011). The ability of WhiA to bind to DNA is mediated by an additional C‐terminal helix‐turn‐helix domain that binds to DNA sequences containing the consensus motif GACAC (Kaiser and Stoddard, 2011; Bush *et al.*, 2013). *In vitro*, WhiA is competent to bind to DNA containing such sequences in the absence of WhiB, but not *in vivo *(Bush *et al.*, 2013). Given that there are ~15,000 GACAC sequences in the *S. venezuelae* genome, but only ~250 are bound by WhiA *in vivo*, it seems likely that the function of WhiB is to tailor the specificity of WhiA, directing its binding to specific promoters *in vivo*. WhiA is constitutively expressed throughout the *Streptomyces* lifecycle, so initiation of sporulation septation is controlled, at least in part, through the developmentally induced expression of its partner protein WhiB (Bush *et al.*, 2017).

In *Corynebacterium, *the orthologue of WhiB, WhcD*_Cg_*, similarly regulates the transcription of genes required for cell division and directly interacts with the orthologue of WhiA *in vitro *(Lee *et al.*, 2017; 2018)*. *WhcD*_Cg_* cannot bind to promoter DNA by itself but stimulates the binding of WhiA to the *ftsZ *promoter, suggesting that WhcD enhances the DNA‐binding activity of WhiA through protein–protein interaction (Lee *et al.*, 2018).

In *Mycobacteria*, WhiB2, the orthologue of WhiB, has been shown to be essential in both *M. smegmatis *and *M. tuberculosis*. Depletion of WhiB2 leads to irreversible filamentous growth and less frequent, aberrant septation (Gomez and Bishai, 2000). Interestingly, WhiB2 is highly similar to the WhiB‐like protein WhiBTM4 from the mycobacteriophage TM4. Apo‐WhiBTM4 competes with apo‐WhiB2 for binding to the *whiB2 *promoter, leading to the downregulation of *whiB2 *expression. Overexpression of WhiBTM4 in *M. smegmatis *gives the same phenotype as the *whiB2 *conditional mutant (Rybniker *et al.*, 2010). In contrast, overexpression of WhiB2 leads to hyperseptation (Gomez and Bishai, 2000). Similar observations have been made in *Streptomyces*, in which overexpression of WhiB leads to hypersporulation (Bush *et al.*, 2017). Collectively, these findings suggest that the WhiB‐orthologues in *Mycobacteria* also function to regulate cell division. Like *whiB2*, the gene encoding the Mycobacterial WhiA orthologue also appears to be essential (DeJesus *et al.*, 2017). It therefore seems likely that WhiA and WhiB function cooperatively in *Mycobacteria*, as well as in *Streptomyces *and* Corynebacteria.*

Given that Wbl proteins are confined to the Actinomycetes, and that WhiA and WhiB seem to function together in these bacteria, it is perhaps surprising to find that WhiA orthologues are prevalent outside the Actinomycetes, being widely distributed in Gram‐positive bacteria. In *Bacillus*, the WhiA orthologue binds to the nucleoid but does not function as a transcription factor, instead regulating cell division and chromosome segregation by an as‐yet‐unknown mechanism (Surdova *et al.*, 2013; Bohorquez *et al.*, 2018). Indeed, the studies in *Streptomyces *and *Corynebacteria* suggest that the ability of WhiA to bind to specific promoters and function as a transcription factor depends upon WhiB, orthologues of which are restricted to the Actinomycete family, and therefore, absent from *Bacillus sp.*

## Class III (WhiB3/WhiD/WhcB)

Like other members of the Wbl family, WhiB3*_Mtb_* has been shown to carry an O_2_ and NO‐sensitive [4Fe–4S] cluster (Singh *et al.*, 2007). Expression of *whiB3 *is increased in the presence of NO and under O_2_‐deficient conditions, suggesting that *M. tuberculosis *might regulate *whiB3 *in response to environmental stimuli associated with infection (Steyn *et al.*, 2002; Banaiee *et al.*, 2006; Geiman *et al.*, 2006; Larsson *et al.*, 2012).

WhiB3*_Mtb_* expression is also upregulated under acidic conditions, both *in vitro *and inside macrophages (Geiman *et al.*, 2006; Rohde *et al.*, 2007), implicating WhiB3*_Mtb_* in the response to low pH within phagosomes. This pH‐dependent increase in *whiB3 *expression is mediated by the response regulator PhoP (Feng *et al.*, 2018). The ability of *M. tuberculosis *to survive conditions of acidic stress within host macrophages is a key feature of persistence during infection. In line with this, deletion of *whiB3 *leads to decreased intraphagosomal survival of *M. tuberculosis *at low pH (Mehta *et al.*, 2016). WhiB3*_Mtb_* has been shown to mediate redox homeostasis in the phagosome by upregulating the biosynthesis of surface‐associated lipids (Singh *et al.*, 2009), by downregulating genes involved in the innate immune response, and by blocking phagosomal maturation (Mehta *et al.*, 2016).

In common with most other Wbls, WhiB3*_Mtb_* lacks a recognisable DNA‐binding motif and, like many of its paralogues, it also interacts with the primary sigma factor, SigA (Steyn *et al.*, 2002). *In vitro, *holo‐WhiB3*_Mtb_* binds DNA weakly in both oxidised and reduced states but the oxidised (disulphide form) of apo‐WhiB3 binds DNA very strongly. Thus, the activity of WhiB3*_Mtb_* as a transcription factor may be controlled by a thiol‐based redox switch (Singh *et al.*, 2009). Under oxidising conditions associated with active infection, apo‐WhiB3*_Mtb_* is likely to be transcriptionally active, whereas under reducing conditions associated with latency, holo‐WhiB3*_Mtb_* is likely to be transcriptionally inactive (Green *et al.*, 2014).

The WhiB3 orthologue in *Streptomyces*, WhiD, was the first Wbl protein shown to carry an [4Fe–4S] cluster (Jakimowicz *et al.*, 2005; Crack *et al.*, 2009). WhiD is one of three Wbl proteins in *Streptomyces *that play a role in the transition from vegetative to reproductive growth that ultimately leads to the formation of spores (Fig. 1; Flärdh and Buttner, 2009; Bush *et al.*, 2015). A *whiD* mutant forms irregularly sized spores that are more sensitive to heat, prone to lysis and show variability in cell wall deposition (Molle *et al.*, 2000). This suggests that WhiD, either directly or indirectly, regulates the expression of genes involved in the later stages of sporulation in *Streptomyces*. Furthermore, the [4Fe–4S] cluster is required for the activity of WhiD*_Sc_* – substitution of any one of the conserved cysteine residues gives a null mutant phenotype (Jakimowicz *et al.*, 2005). Like WhiB1 of *M. tuberculosis *(see above), the [4Fe–4S] cluster of WhiD*_Sc_* reacts with eight molecules of NO. This nitrosylation is extremely rapid, with the reaction occurring 10^4^ times faster than that with O_2_ (Crack *et al.*, 2011). However, as is the case for its mycobacterial orthologue, the physiological significance of the NO‐sensitivity of WhiD has yet to be established.

In *Corynebacterium*, the WhiB3/WhiD orthologue, WhcB, has not been extensively studied. WhcB*_Cg_* is expressed during stationary phase and has been suggested to have a role in controlling the expression of gene(s) in the oxidative stress response pathway (Lee *et al.*, 2012).

## Class IV (WhiB4/WblA/WhcA)

In *M. tuberculosis*, WhiB4 has been shown to regulate the response to oxidative stress to modulate virulence. Transcriptional analysis identified WhiB4*_Mtb_*‐repressed genes that are differentially expressed under various stress conditions. Deletion of *whiB4 *leads to hyper‐induction of antioxidants, increased resistance to oxidative stress *in vitro *and enhanced survival in macrophages, reflecting the importance of WhiB4*_Mtb_* in maintaining redox homeostasis (Chawla *et al.*, 2012). As has been shown for other Wbl‐family members, WhiB4 contains an O_2_‐ and NO‐sensitive [4Fe–4S] cluster (Alam *et al.*, 2007; Chawla *et al.*, 2012). However, the [4Fe–4S] cluster of WhiB4*_Mtb_* appears to be more sensitive to O_2_ than reported for other Wbls, such as WhiB3/WhiD (Singh *et al.*, 2007; Crack *et al.*, 2009) and WhiB1(Kudhair *et al.*, 2017), suggesting a specific role for WhiB4*_Mtb_* in redox‐sensing (Chawla *et al.*, 2012). In line with this, holo‐WhiB4*_Mtb_* is not competent to bind to DNA but the loss of the [4Fe–4S] cluster and oxidation of the coordinating cysteines strongly stimulates DNA binding. *In vitro *and in isolation, oxidised apo‐WhiB4 does not appear to bind to DNA in a sequence‐specific manner but rather preferentially binds to GC‐rich sequences in the minor‐groove. Oligomerisation of WhiB4*_Mtb_*, driven by disulfide bond formation, has been observed both *in vitro *and *in vivo *in *M. smegmatis*, suggesting a possible mechanism for the control of WhiB4*_Mtb_* activity (Chawla *et al.*, 2012).

The WhiB4 orthologue, WblA, is one of three Wbl‐family members that regulate differentiation in *Streptomyces*. Whereas WhiB and WhiD regulate the initiation of sporulation septation and spore maturation, respectively, WblA appears to play an earlier role in development, during the formation of aerial hyphae (Fig. 1). In *S. coeilcolor*, a *wblA *mutant exhibits a defect in sporulation, with some aerial hyphae failing to sporulate and appearing thinner compared to wild‐type (Fowler‐Goldsworthy *et al.*, 2011). Microarray analysis indicates that WblA influences the expression of genes involved in antibiotic production, morphological differentiation and oxidative stress (Kang *et al.*, 2007; Kim *et al.*, 2012a; Yu *et al.*, 2014). However, WblA binding to the promoters of target genes has not yet been demonstrated, either *in vivo *(e.g. by ChIP‐seq) or *in vitro *(e.g. by EMSA).

Like *M. tuberculosis *WhiB4 and *S. coelicolor *WblA, the *Corynebacterium* orthologue, WhcA, appears to negatively regulate the expression of genes involved in the response to oxidative stress (Choi *et al.*, 2009). WhcA directly interacts with SpiA (Stress protein interacting with WhcA, annotated as a dioxygenase/oxido‐reductase and suggested to be involved in signal perception) and this interaction can be disrupted in the presence of the oxidant diamide (Park *et al.*, 2011; 2012). This mechanism appears to be conserved in *Streptomyces*, in which a SpiA orthologue has been identified and similarly demonstrated to be a negative regulator of the WblA‐dependent oxidative stress response (Kim *et al.*, 2013). However, in *Mycobacteria*, there is no clear SpiA‐orthologue nor have other protein partners of WhiB4 been identified.

## Class V (WhiB7/WblC)

WhiB7 and its orthologue, WblC, control innate multi‐drug resistance in *Mycobacteria *and *Streptomyces *respectively (Morris *et al.*, 2005; Ramon‐Garcia *et al.*, 2013). There is no obvious orthologue in *Corynebacteria *(Table 2 and Fig. 3). In *M. tuberculosis*, expression of *whiB7 *is induced by exposure to sub‐inhibitory concentrations of translation‐inhibiting antibiotics, such as erythromycin, streptomycin and tetracycline (Morris *et al.*, 2005). As well as controlling its own expression, WhiB7*_Mtb_* has been shown to control the expression of a plethora of antibiotic resistance genes (Morris *et al.*, 2005; Burian *et al.*, 2013). In this way, induction of WhiB7*_Mtb_* by a specific antibiotic can give rise to a broad spectrum of resistance. Overexpression of *whiB7 *promotes multi‐drug resistance in *M. tuberculosis*, making WhiB7*_Mtb_* an attractive potential target for new therapeutics (Morris *et al.*, 2005). In addition to antibiotics, *whiB7 *is also upregulated in response to fatty acids that may be accumulated internally or encountered within eukaryotic hosts during infection by *M. tuberculosis *(Morris *et al.*, 2005).

In *Streptomyces*, WblC protein levels are induced in the presence of translation‐inhibiting antibiotics. How this induction occurs is not known, but it may well involve the extremely long 5′ UTR of the *wblC* mRNA, which appears to contain an ORF encoding a small protein (Yoo *et al.*, 2016). Recently, the first direct target of WblC*_Sc_* was characterised (Yoo *et al.*, 2016). Unexpectedly, this target was the *sigR‐rsrA* operon, encoding the sigma factor SigR and its redox‐sensitive antisigma factor, RsrA, which together control the principal oxidative stress response in *Streptomyces *(Paget *et al.*, 1998; Kang *et al.*, 1999). Under oxidative stress conditions, SigR is liberated from RsrA and activates transcription of a large regulon of genes that help the bacterium survive oxidative stress and re‐establish normal redox poise (Paget *et al.*, 1998; 2001; Kim *et al.*, 2012b). This rapid and transient response depends upon the autoregulatory function of SigR that induces expression of a longer (but unstable) isoform of SigR (SigR′) from a SigR‐target promoter (*sigRp2*) (Kim *et al.*, 2009). In contrast, in the presence of antibiotics, WblC*_Sc_* mediates a longer‐lasting response by binding to a more downstream (and SigR‐independent) promoter, *sigRp1*, thereby increasing the expression of the shorter (but stable) isoform of SigR (Yoo *et al.*, 2016).

This work showed that activation of the SigR regulon not only increased resistance to oxidative stress, but also resistance to translation‐inhibiting antibiotics, revealing a perhaps unexpected overlap between the types of cellular damage caused by oxidative stress and by the inhibition of translation (Yoo *et al.*, 2016). It seems likely that the transient expression of the regulon mediated by the unstable isoform SigR′ is sufficient to cope with oxidative stress, but the more sustained expression of the regulon mediated by the stable SigR isoform is required to deal with the consequences of the inhibition of ribosomes (Yoo *et al.*, 2016). In *Mycobacteria, *the SigR homologs (SigE*_Mtb_* and SigH*_Mtb_*) are also likely to be targets of WhiB7*_Mtb_* since expression of both also increases upon addition of translation‐inhibiting antibiotics (Yoo *et al.*, 2016). In both *Streptomyces* and *Mycobacteria*, this SigR‐dependent shift to reducing conditions would be expected to play a role in maintaining the [4Fe–4S] cluster of WhiB7/WblC, meaning that WhiB7/WblC would function in an autoregulatory loop. In line with this, treatment of *M. smegmatis *with erythromycin creates a highly reducing environment in the cytoplasm and this shift is dependent upon *whiB7 *(Burian *et al.*, 2012; 2013).

Of all the Wbls, the way in which WhiB7/WblC binds DNA is most clear. This is because WhiB7/WblC is the only member of the Wbl family that carries a recognised DNA‐binding motif, the AT‐hook (Fig. 2). AT‐hooks do not bind to specific consensus sites but rather bind to the minor groove of AT‐rich DNA. They are often found in proteins that carry additional DNA‐binding domains, suggesting that their function is to fine‐tune the specificity or affinity of DNA binding (Aravind and Landsman, 1998). In common with many other Wbls, holo‐WhiB7*_Mtb_* interacts with the primary sigma factor in *M. tuberculosis, *SigA*. *WhiB7*_Mtb_* ‐mediated multi‐drug resistance is dependent both on the holo‐WhiB7‐SigA interaction and on the AT‐rich binding site of WhiB7*_Mtb_*, which is found just upstream of the ‐35 element bound by SigA in *M. tuberculosis*. Therefore, it is likely that in the presence of antibiotics, the function of WhiB7/WblC is to recruit the primary sigma factor to a specific subset of promoters to induce the expression of resistance genes (Burian *et al.*, 2013).

## Discussion

Despite the fact that Wbl proteins regulate key processes across the Actinobacteria, including virulence, antibiotic resistance and morphogenesis, for many years their biochemical function has remained elusive. In the past, it has been suggested that Wbls can function as disulphide reductases (Alam *et al.*, 2007; Garg *et al.*, 2007) or chaperones (Konar *et al.*, 2012). However, given the many studies summarised in this review, it seems certain that WhiB‐like proteins function primarily to regulate transcription. *In vitro *studies, mostly in *Mycobacteria*, reveal the ability of Wbls to bind to DNA and *in vivo* ChIP‐seq in *Streptomyces *confirms that WhiB, and therefore, likely all Wbls, function to regulate transcription (Bush *et al.*, 2016). Recently, induced expression of transcription factors followed by ChIP‐seq has been coupled to transcriptional profiling in *M. tuberculosis *to identify genome‐wide DNA‐binding events (Rustad *et al.*, 2014; Minch *et al.*, 2015). Careful analysis of these data and similar experimental approaches are needed to further reveal the regulatory roles of Wbl proteins *in vivo*. Much of the original uncertainty regarding the biochemical function of Wbls arose from the absence of a recognisable DNA‐binding domain. WhiB7/WblC is the only exception, containing an AT‐hook, a motif that typically binds AT‐rich sequences in the minor groove (Aravind and Landsman, 1998). The other Wbls contain a series of positively charged amino acids at varying frequencies towards the C‐terminus (Fig. 2). Given this, it is hard to understand how such proteins in isolation could bind specific target promoters *in vivo*. In line with this, several studies suggest that binding to DNA *in vitro *is non‐specific (Chawla *et al.*, 2012)*, *or else occurs with a low‐degree of sequence discrimination (Singh *et al.*, 2009) or to a large promoter region with no core motif (Smith *et al.*, 2010). An emerging theme, therefore, is that the ability of Wbls to function as site‐specific transcription factors seems to depend on partner or accessory proteins.

In *Mycobacteria*, WhiB1, WhiB3 and WhiB7 have all been shown to interact directly with the primary sigma factor SigA. In the case of WhiB7*_Mtb_*, the AT‐hook mediates binding to an AT‐rich sequence upstream of the SigA ‐35 element, likely increasing the specificity of the sigma factor at a subset of promoters (Burian *et al.*, 2013). The interactions between SigA and WhiB1/WhiB3/WhiB7 have all been shown to occur via region 4 of the sigma factor (Steyn *et al.*, 2002; Burian *et al.*, 2013; Kudhair *et al.*, 2017), the domain responsible for contacting the ‐35 element and a well‐established target of Class II activators (Browning and Busby, 2016). For SigA‐WhiB7, the interaction requires a triplet of residues (EPW), adjacent to the conserved GVWGG‐motif of WhiB7*_Mtb _*(Fig. 2) and part of the loop region previously predicted to facilitate protein‐protein interaction (Soliveri *et al.*, 2000; Burian *et al.*, 2013). WhiB3*_Mtb_* contains a similar (EPY) motif, in line with the observed SigA‐WhiB3 interaction (Steyn *et al.*, 2002). For SigA‐WhiB3 and SigA‐WhiB7, the interactions can be eliminated by a specific SigA substitution (R515H) in region 4 (Steyn *et al.*, 2002; Burian *et al.*, 2013). In contrast, in WhiB1*_Mtb_*, the triplet motif of the ‘middle‐domain’ is not conserved (Fig. 2). Furthermore, the R515H SigA variant is viable in *Mycobacteria*, suggesting that WhiB1, which is essential for viability, interacts with SigA via different residues of the Wbl ‘middle‐domain’ and SigA region 4. Similarly, WhiB2_M_ *_tb_*is essential (DeJesus *et al.*, 2017) and carries a different (ERF) triplet of residues adjacent to the GVWGG‐motif (Fig. 2). Therefore, if WhiB2*_Mtb _*also interacts with SigA, it is likely to do so via different amino acid contacts, or else it interacts with a different sigma factor. Indeed, another member of the Wbl family, *M. tuberculosis *WhiB5, does not seem to interact with SigA (Casonato *et al.*, 2012), suggesting that it might interact with an alternative sigma factor. Considering this, it is interesting to note that three of the genes encoding Wbls in *S. coelicolor *(*whiD*, *wblE* and *wblJ*) are located close to sigma factor genes and one *wbl *gene, located on the SCP1 plasmid, encodes a protein (WblP) consisting of a Wbl domain fused to an ECF sigma factor (Bentley *et al.*, 2004). Wbl‐sigma interactions have not been studied in *Streptomyces *and *Corynebacteria*, although it seems likely that the Wbl‐sigma interactions observed in *Mycobacterium *would be conserved between the clear orthologues in *Streptomyces *and *Corynebacteria* discussed in this review (Table 2 and Fig. 3). In this context, it is interesting to note that the triplet of residues adjacent to the G[V/I]WGG‐motif is conserved among orthologues in each of the five classes of Wbl proteins (Fig. 2).

Individual actinobacterial species typically contain multiple WhiB‐like paralogues (Table 1 and Fig. 3), all carrying an [4Fe–4S] cluster (Fig. 2). This raises the question as to how Wbls can maintain distinct regulatory functions in the same cell. In other paralogous families of transcription factors, innate operator DNA sequence discrimination plays a central role. The limited ability of Wbls to function as site‐specific transcription factors (except for WhiB7/WblC) means that the ability to direct a specific cellular response based on innate DNA sequence recognition is at least limited. Clearly, the regulation of *wbl *gene expression plays an important role. In *Mycobacteria *and *Corynebacteria*, the expression of different *wbl *genes has been shown to be activated by various stress conditions (e.g. low nutrient availability, heat, oxidative stress) (Geiman *et al.*, 2006; Lee *et al.*, 2013) and, in both *Mycobacteria *and *Streptomyces, *transcription of *whiB7/wblC* has been shown to be activated by translation‐inhibiting antibiotics (Morris *et al.*, 2005; Yoo *et al.*, 2016). In *Streptomyces*, the expression of developmental genes, including *whiB*, *whiD* and *wblA*, is controlled as part of a complex regulatory network (Flärdh and Buttner, 2009; Bush *et al.*, 2015; 2017). The overall effect of such control is that few Wbl paralogues are likely to be present at the same time in the cell. This partly ensures that the correct Wbl‐mediated transcriptional response is mounted on exposure to a given signal or stress.

As highlighted in this review, another key contributory factor may be the need for Wbls to interact with partner proteins. Several Wbl paralogues have been shown to interact with the primary sigma factor, SigA. Other protein partners for Wbls have also been identified; SpiE interacts with WhcE in *Corynebacterium* (Park *et al.*, 2016) and SpiA interacts with WhcA/WblA in *Corynebacterium *and *Streptomyces *(Park *et al.*, 2012; Kim *et al.*, 2013). In *Streptomyces*, WhiB has been shown to co‐control the expression of genes required for sporulation with an unrelated protein, WhiA. One of the consequences of Class II activation is that the α‐CTDs of the RNAP holoenzyme are unable to bind in their preferred positions, upstream of the ‐35 promoter element. This means that additional transcription factors, so‐called Class I activators, that bind further upstream and interact with the α‐CTDs, can also influence transcription, creating synergy in bacterial regulatory networks (Browning & Busby, 2016). It is tempting to speculate that WhiB functions as a Class II activator to bind and recruit a sigma factor, perhaps SigA (called σ^HrdB^ in *Streptomyces*), while WhiA binds further upstream akin to a Class I activator. It may therefore be a WhiA‐WhiB‐SigA tripartite complex that regulates gene expression to initiate sporulation septation in *Streptomyces*. Further research is required to establish whether other members of the Wbl family function in concert with partner proteins and what the functional consequences of such interactions are.

Interestingly, the presence and status of the [4Fe–4S] cluster seems to influence DNA‐binding in different ways across the Wbl family. The cluster appears to be required for *S. venezuelae *WhiB and *M. tuberculosis *WhiB7 to bind DNA (Burian *et al.*, 2013; Bush *et al.*, 2016), whereas the mycobacterial apo‐WhiB1, apo‐WhiB2, apo‐WhiB3 and apo‐WhiB4 all bind DNA, at least *in vitro *(Singh *et al.*, 2009; Rybniker *et al.*, 2010; Smith *et al.*, 2010; Chawla *et al.*, 2012; Stapleton *et al.*, 2012; Kudhair *et al.*, 2017). In the case of apo‐WhiB3*_Mtb_* and apo‐WhiB4*_Mtb_*, the oxidation state of the coordinating cysteine residues appears important – the oxidised, disulphide forms have increased affinity for DNA (Singh *et al.*, 2009; Chawla *et al.*, 2012). Despite these findings, it cannot be ruled out that individual Wbls have different regulatory roles depending upon the state of the [4Fe–4S] cluster. For example, WhiB6 performs such a dual function in *Mycobacteria*, differentially regulating the expression of two sets of genes (Chen *et al.*, 2016). Further *in vivo* identification of the genes under the direct control of Wbls is required to grasp the full scope of Wbl‐mediated regulation of gene expression.

The function of Wbl proteins may also be controlled in part by their relative sensitives to O_2_ and NO. Although Wbls all contain a [4Fe–4S] cluster, some are relatively tolerant of aerobic conditions, such as WhiB1*_Mtb_* (Kudhair *et al.*, 2017), whereas others are more sensitive to O_2_, such as WhiB4*_Mtb_* (Chawla *et al.*, 2012). In the case of WhiB1*_Mtb_*, the aerobic stability of the protein further strengthens the hypothesis that NO, and not O_2_ controls its activity. In *Streptomyces*, the [4Fe–4S] cluster of WhiD reacts several orders of magnitude more rapidly with NO than with O_2_ (Crack *et al.*, 2011). Although the reaction with NO *in vitro *may be a conserved feature of Wbls, it seems unlikely that all Wbl proteins respond to NO *in vivo, *given their diverse biological roles. The ability of some Wbls to respond to NO makes sense in pathogenic *Mycobacteria *and *Corynebacteria*, which encounter NO as part of the host defence response. *Streptomyces *encounter endogenously produced NO (Johnson *et al.*, 2008; Sasaki *et al.*, 2016) and, like many other bacteria, use a dedicated NO sensor (NsrR) that controls the expression of detoxifying enzymes (Crack *et al.*, 2015). However, further research is required to understand the *in vivo *significance of NO sensing in the Wbl family. Indeed, the [4Fe–4S] cluster of Wbl proteins is likely to be sensitive to other reactive oxygen species (Crack *et al.*, 2009) and reactive nitrogen species, and major future challenges are to decipher not only the network of protein–protein interactions through which Wbl proteins effect their functions, but also the precise nature of the cellular signal(s) to which they respond.

## Note

Whilst this review was under consideration, Chawla *et al.*, reported that WhiB4Mtb dynamically couples genome condensation to the oxidative stress response in *Mtb *(Chawla, M., Mishra, S., Anand, K., Parikh, P., Mehta, M., Vij, M., Verma, T., Singh, P., Jakkala, K., Verma, H.N., AjitKumar, P., Ganguli, M., Narain Seshasayee, A.S., Singh, A. 2018. **19**: 116‐133). Extending their work reported in this review, the authors demonstrate, *in vitro*, that under conditions of oxidative stress, disulphide‐linked oligomerisation of WhiB4Mtb stimulates the protein’s ability to condense DNA. In line with these findings, *in vivo *ChIP‐seq coupled to microarray transcriptional profiling revealed non‐specific binding of WhiB4Mtb to GC‐rich genomic regions leading to both direct and indirect effects on transcription. These findings suggest that WhiB4Mtb links the oxidative stress response of *Mtb *to DNA organisation and gene expression.
